# Assessment of eHealth Literacy in Healthcare Service Users: Construction and Validation of a Measurement Instrument

**DOI:** 10.2174/0117450179393541250722062947

**Published:** 2025-07-29

**Authors:** Juan Morales, César Augusto Eguia

**Affiliations:** 1 University of Sciences and Humanities (UCH), e-Health Research Center, Lima, Peru; 2 University of Sciences and Humanities (UCH), Faculty of Health Sciences, Lima, Peru

**Keywords:** Digital Health, Health Literacy, Health Services, Patients, Surveys and Questionnaires, Peru

## Abstract

**Introduction:**

eHealth literacy is influenced by Internet access and is associated with health status. The aim of this study was to develop and validate an instrument called eHealth-Much to measure eHealth literacy in users of healthcare services.

**Methods:**

An instrumental research design was used. Content validity was assessed by expert judgment and quantified using Aiken’s V coefficient. A polychoric correlation matrix was used for the items. Sample adequacy was assessed through the Kaiser-Meyer-Olkin (KMO) index and Bartlett's test of sphericity before conducting exploratory factor analysis (EFA). The EFA was conducted using the Weighted Least Squares (WLS) extraction method with Oblimin rotation. Reliability was assessed using Cronbach’s Alpha (α) and McDonald’s Omega (ω) coefficients.

**Results:**

Twelve experts from five different countries participated in the content validation process, obtaining a V coefficient of 0.93 (95% CI: 0.79–0.97). A total of 1,068 health service users of both sexes participated in the construct validity testing. The median age was 32 years (IQR: 13, Q1: 26, Q3: 39; Min: 17, Max: 78). The KMO index was 0.92 (Bartlett's test, p < 0.001). The EFA suggested four factors labeled “Digital Literacy” (WLS1), “Digital Self-Management” (WLS4), “Digital Skill” (WLS3), and “Scientific Empowerment” (WLS2), which together explained 59.3% of the total variance. The reliability coefficients obtained were 0.86 for Cronbach’s alpha and 0.90 for McDonald’s omega. Percentile ranks and normative scores were also established for the sample.

**Discussion:**

The factorial structure obtained theoretically supports the multidimensionality of the construct, aligning with previous models of digital health literacy.

**Conclusion:**

The scale demonstrates adequate levels of validity and reliability. It may be considered a viable option for use in both primary care settings and hospital environments. Further studies are recommended to expand the psychometric analysis.

## 1 INTRODUCTION

Currently, 67% of the world's population uses the Internet, fundamentally transforming human interactions [1]. Digital connectivity is essential for daily life, providing access to critical services and educational opportunities [2].

Information and communication technologies (ICT) are essential for modern healthcare [3]. The Internet has become a primary source of health information for patients [4-6].

Health literacy refers to an individual's ability to access, process, and comprehend basic health information and services necessary for making informed health decisions [7]. E-health literacy builds upon this concept, emphasizing the ability to navigate, evaluate, and apply health information from digital sources to address or resolve health-related issues [8].

E-health literacy is strongly influenced by exposure to digital technologies, Internet access, and online health information sources [9, 10]. Higher e-health literacy is observed (AOR = 2.35; 95% CI: 1.67, 3.30) among individuals with Internet access [11], as well as those with access to smartphones or other mobile devices, and those with higher levels of education [12].

E-health literacy is associated with better self-care, improved medication adherence, and enhanced disease management and preventive behaviors [13]. Low health literacy is linked to adverse health outcomes and suboptimal utilization of health services [14]. Moreover, e-health literacy has a positive impact on health promotion, disease prevention, psychological well-being, social support, and healthcare service utilization [15].

A widely recognized and utilized instrument for assessing digital literacy is the eHealth Literacy Scale (eHEALS) [16]. The eHEALS was originally designed for the first generation of eHealth technologies (Web 1.0), with an emphasis on information retrieval [17, 18]. However, it lacks comprehensiveness in assessing eHealth literacy within the context of Web 2.0 [15]. The World Wide Web (WWW) has evolved from Web 1.0, which was characterized by static, information-centered content, to Web 2.0, which introduced interactivity and user-generated content, giving rise to social media platforms. It has continued to evolve through Web 3.0 and Web 4.0, and is expected to advance further toward Web 5.0 in the future [19].

The development of a new measurement instrument is justified for several reasons. First, due to the obsolescence of existing tools [20]; moreover, the interactive applications that characterize Web 2.0 and beyond require a broader range of skills [17]. Currently available instruments present limitations resulting from their age, which compromises their relevance in a context where digital environments have rapidly evolved. This is the case of the eHEALS [16], one of the most widely used scales for measuring eHealth literacy, which was developed prior to the rise of social media and mobile web technologies [21], and has proven insufficient to assess the new skill sets required for interacting with emerging technologies. In the Peruvian context and across different geographical settings, both surveyors and healthcare users have perceived shortcomings [22-24].

Second, since the onset of the COVID-19 pandemic [25], the digitalization of individuals and electronic environments became essential to ensure healthcare delivery and continuity of services [26]. In-person consultations were replaced by telemedicine in both primary care centers and hospitals [27]. Moreover, telemedicine and eHealth have demonstrated significant potential for improving health outcomes [26].

Social media can be used for various health-related purposes, with both healthcare organizations and patients maintaining an active presence, giving rise to new dynamics in the access, search, use, and dissemination of health information [28-30]. These changes demand tools that assess not only basic digital competencies, but also other dimensions such as interaction in digital environments, information quality, and informed decision-making. Therefore, the objective of this study was to develop and validate a contextualized and updated instrument to assess eHealth literacy among healthcare service users, thereby contributing to a better understanding and approach to health-related issues.

## 2 MATERIALS AND METHODS

### 2.1 Approach and Type of Study

The study adopted a quantitative [31, 32] and methodological approach [33, 34]. Since the research focused on the development, design, validation, and application of a measurement instrument, it can be classified as instrumental research [35].

### 2.2 Study Scope, Population, and Sample

This study was conducted in the district of “*Mi Perú*”, located in the Callao Region, which has a population of approximately 60,000 inhabitants [36]. The participants were users of healthcare services from a public health facility in that district.

Individuals of both sexes aged 18 years or older who attended outpatient consultations and provided informed consent to voluntarily participate in the study were included. Participants with evident cognitive impairments, communication difficulties, or who failed to adequately complete the data collection instrument were excluded.

To obtain a referential sample (n), we estimated it using the following equation [37]: n = [DEFF_*_Np (1-p)]/ [(d2/Z21-α/2_*_(N-1) + p_*_(1-p)], where: N: 60000, p: 50%, d: 5%, and EDFF=1.

For a 95% confidence interval (95% CI), the minimum estimated sample size was 382 subjects. However, there is no universally established minimum sample size for instrument validation studies [38].

Validation studies typically suggest ranges of 5 to 15 respondents per item [39, 40], item-response ratios from 1:3 to 1:20, and sample sizes exceeding 1,000 as excellent [41]. For this study, the minimum required sample size was calculated as 15 respondents per item in the initial questionnaire. However, to ensure greater representativeness, we recruited a total of 1,068 participants using probability sampling methods.

### 2.3 General Description of Study

**Fig. (1):**
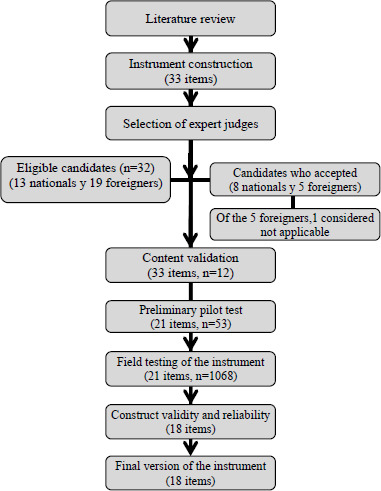
General description of the study.

The study was conducted in the following stages: literature review, instrument construction, selection of expert judges, content validation, preliminary pilot test, field testing of the instrument, construct validity, and reliability assessment (Fig. **1**). The entire study process took place from January to December 2024.

#### 2.3.1 Literature Review

A literature search was conducted in PubMed using eHealth Literacy Scale' as the search term. The instrument was designed based on Norman's Lily model and the eHealth Literacy Scale (eHEALS) [8, 16].

#### 2.3.2 Instrument Construction

A key aspect in instrument development is ensuring that all domains of the variable of interest are adequately represented [20]. In this study, we followed the 'Lily model' of eHealth literacy [8], which includes six types of literacy: Traditional Literacy, Information Literacy, Media Literacy, Health Literacy, Computer Literacy, and Scientific Literacy. Items were developed for each of these domains accordingly.

The initial instrument was designed following Deming’s plan-do-check-act (PDCA) cycle [42]. A total of 33 items were proposed, distributed across six domains: Traditional Literacy (4 items), Information Literacy (4 items), Media Literacy (6 items), Health Literacy (7 items), Computer Literacy (7 items), and Scientific Literacy (5 items). This set of items constituted the initial version of the instrument (Version 1).

#### 2.3.3 Selection of Expert Judges

Professionals with postgraduate studies, as well as teaching and clinical experience, were considered. Experience in eHealth was considered desirable. Candidates were selected based on their published articles or their participation in an eHealth academic program. The introduction letter and collaboration request were sent via email, using addresses retrieved from their publications or through their social and academic networks. Upon acceptance, they received the validation documents.

A total of 36 candidates were invited via email, of whom 13 experts from Bolivia, Ecuador, Spain, Mexico, and Peru responded and participated in the validation process. Of the two judges from Ecuador, one was excluded as their evaluation was deemed inapplicable; however, their suggestions were taken into account. The final panel for validation consisted of 12 judges. The literature suggests that a panel of at least 10 experts provides a reliable estimate of an instrument’s content validity [43].

#### 2.3.4 Content Validation

For content validity assessment, the criteria of appropriateness, relevance, and clarity were examined. These criteria were assessed using a Likert scale ranging from 1 to 4, with the following categories: Does not meet the criteria (1 point), Low level (2 points), Moderate level (3 points), and High level (4 points). Additionally, each item included options for suggestions and comments.

Content validity was assessed using Aiken's V coefficient, which quantifies the degree of consensus among judges. This coefficient ranges from 0 to 1, with higher values indicating greater agreement [44, 45]. A value above 0.8 was considered indicative of strong consensus [44, 45].

In addition to the statistical analysis from the first evaluation, necessary modifications were made in response to feedback, resulting in a new version. Before undergoing the preliminary pilot test, the instrument was reviewed and approved by experts in instrument design and validation (Version 2).

#### 2.3.5 Preliminary Pilot Test

Before testing the questionnaire with the target respondents, it is recommended to conduct a pilot test on a small sample of 30 to 50 respondents [46, 47]. The pilot test can be both qualitative and quantitative [20]. This preliminary test helps identify ambiguous items and gather suggestions for potential improvements [46, 48].

For this purpose, we used the questionnaire in digital format (Version 2), developed in Google Forms®, and administered it to a sample with characteristics comparable to those of the target population.

A total of 53 healthcare service users (43 females, 10 males) were randomly selected, with a mean age of 35.9 years (SD: 10.9; Min: 18, Max: 64). The completion of the instrument took approximately 10 to 15 minutes. Additionally, participants' suggestions and observations regarding clarity and simplicity were collected and considered in developing the final version of the instrument.

In this test, priority was given to the qualitative component, which allowed for the identification of difficulties in comprehension, application time, and data collection feasibility. Based on these findings, the questionnaire was refined and improved, resulting in the final version (Version 3), which was used for the main validation. The final version consisted of 21 items (q1 to q21), rated on a Likert scale from 1 to 5, with the following scale: 1 = strongly disagree, 2 = somewhat disagree, 3 = neither agree nor disagree, 4 = somewhat agree, and 5 = strongly agree.

#### 2.3.6 Field Testing of the Instrument

At this stage, the final version of the questionnaire (Version 3) was administered to a sample of the target population. A total of 1068 randomly selected healthcare service users of both sexes participated.

Data collection was conducted mainly through interviews and, to a lesser extent, through self-administered questionnaires. The instrument was administered across different services of a public healthcare facility in the district of 'Mi Perú' by two healthcare personnel who had received prior training. They conducted the application for at least 180 minutes per session. Data collection took place from September to October 2024.

#### 2.3.7 Construct Validity and Reliability

The following procedures were performed on the data collected during the field test: the correlation of each questionnaire item was assessed. To determine whether to retain or remove items, an item-total analysis was conducted. Items with item-total correlations below 0.3 were removed [49, 50].

A polychoric correlation matrix of the items was generated, along with its graphical representation. Before conducting the Exploratory Factor Analysis (EFA), sample adequacy was assessed using the Kaiser-Meyer-Olkin (KMO) index and Bartlett's test of sphericity. The KMO index ranges from 0 to 1. A value above 0.7, along with a statistically significant Bartlett's test of sphericity (p<0.05), is recommended to proceed with EFA [51].

In the EFA, the optimal number of factors to extract was determined using the Kaiser rule (Eigenvalue >1) and the scree plot, the most commonly used methods [52]. Additionally, parallel analysis was performed, as it is considered highly reliable [53].

The questionnaire used in this study comprised ordinal variables (Likert-type items). Ordinal variables do not satisfy the assumptions of linearity and normality [39]. When normality is unlikely or when univariate skewness and kurtosis are excessively high, more robust correlation methods, such as polychoric correlation, are recommended [39]. Assuming a normal distribution of latent variables, polychoric correlations provide more precise estimates that are less affected by measurement error, outperforming Pearson and Spearman correlations in the analysis of ordinal data [54, 55].

For this study, EFA was performed on the polychoric correlation matrix, using Weighted Least Squares (WLS) extraction with Oblimin rotation. WLS with polychoric correlations generally outperforms Maximum Likelihood (MLE) with Pearson correlations. While MLE tends to underestimate true factor loadings, WLS may overestimate them [54].

Instrument reliability was assessed using Cronbach's alpha coefficient. Cronbach's alpha is commonly used to assess internal consistency [56-58]. An alpha value between 0.70 and 0.95 is considered acceptable for reliability assessment [56].

### 2.4 Statistical Analysis

The dataset automatically generated in Google Forms® was downloaded as a Microsoft Excel® spreadsheet, reviewed, recoded, and imported into RStudio (version 4.2.3). All statistical procedures, tables, and graphs were conducted using this software and later edited in Microsoft Excel®.

Aiken's V coefficient and its 95% confidence interval (95%CI) were calculated using the following formula: V= (X-l)/k, L= {2nkV+Z2-Z[4nkV(1-V) + Z^2^]1/2} ÷ [2(nk+Z2)], U={2nkV+Z2+Z[4nkV(1-V) + Z^2^]1/2} ÷ [2(nk+Z2)]. Where: X: Average of grades, l: minimum grade, k: range of grades (max.-min.), L: 95% lower limit, U: 95% upper limit [44, 45].

A descriptive analysis was conducted on the preliminary pilot test and the field test data of the instrument. The feasibility of the EFA was assessed using the Kaiser-Meyer-Olkin (KMO) index and Bartlett's test of sphericity. Factor extraction was performed using Weighted Least Squares (WLS) with Oblimin rotation. Reliability was assessed through Cronbach's alpha coefficient (α) and, for comparison, McDonald's omega coefficient (ω).

### 2.5 Ethical Aspects

The ethical principles outlined in the Declaration of Helsinki of the World Medical Association [59] were followed. Participation was voluntary, without any financial or material incentive, and written informed consent was obtained. Formal authorization was also obtained from the head of the healthcare facility. The research protocol was reviewed and approved by the Ethics Committee of the University of Sciences and Humanities, Lima, Peru.

## 3 RESULT

### 3.1 Content Validation

Twelve experts from five different countries participated in the content validation, resulting in an overall Aiken's V coefficient of 0.93 (95% CI: 0.79–0.97). For the domains of relevance, pertinence, and clarity, the Aiken's V coefficients were 0.94, 0.94, and 0.92, respectively. Items with coefficients below 0.8 were revised, while those with high validity coefficients were either retained, refined, or modified according to the experts' observations (Appendix **1**).

### 3.2 Field Testing of the Instrument

This test employed the final 21-item version (q1 to q21). A total of 1,068 healthcare users participated, with a median age of 32 years (IQR: 13; Q1: 26; Q3: 39; range: 17-78). The sample consisted of 86.3% women (n=922) and 13.7% men (n=146).

Participants provided ratings from 1 to 5, with some items showing skewness (absolute value >1) and leptokurtic distribution (kurtosis >3) (Appendix **2**).

### 3.3 Construct Validity

A preliminary feasibility analysis for Exploratory Factor Analysis (EFA) was conducted using data from all 21 items, estimating the Kaiser-Meyer-Olkin (KMO) measure and Bartlett's test of sphericity (KMO=0.91, Bartlett's p<0.001). Internal reliability assessment for each factor was performed using Cronbach's alpha coefficient, which led to the exclusion of items with unacceptable or questionable values (items q1, q7, and q11 were removed), resulting in an 18-item instrument (Q1 to Q18). The final EFA was performed based on the polychoric correlation matrix of the remaining 18 items (Appendix **3**).

### 3.4 Exploratory Factor Analysis

**Fig. (2):**
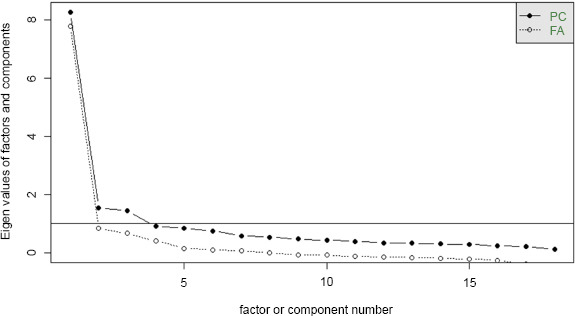
Scree Plot for Factor Retention Based on WLS estimation.

The scree plot visualization based on the eigenvalue-greater-than-one rule suggested a three-factor solution (Fig. **2**). For the sample adequacy measures of the 18 items, we calculated the Kaiser-Meyer-Olkin (KMO) measure and Bartlett's test of sphericity (KMO = 0.92, Bartlett's p < 0.001) (Table **1**).

Using the Weighted Least Squares (WLS) extraction method with Oblimin rotation, we identified four factors. Items were grouped as follows: WLS1 (Q7, Q8, Q9, Q10, Q11, Q12, Q13), WLS4 (Q1, Q2, Q3, Q4, Q5), WLS3 (Q14, Q15, Q16), and WLS2 (Q6, Q17, Q18) (Table **2**).

These four factors collectively accounted for 59.3% of the total variance (WLS1: 22.4%; WLS4: 15.7%; WLS3: 11.5%; and WLS2: 9.8%) Table **3**.

### 3.5 Measures of Reliability

Reliability analysis for the full scale yielded a Cronbach's alpha coefficient of 0.86 (95% CI: 0.85-0.87). The Cronbach's alpha coefficients for each factor were: WLS1 (0.81, 95% CI: 0.80-0.83), WLS4 (0.72, 95% CI: 0.70-0.75), WLS3 (0.67, 95% CI: 0.64-0.71), and WLS2 (0.60, 95% CI: 0.56-0.64). Additionally, McDonald's total omega coefficient was 0.90 Table **4**.

Cronbach's alpha coefficients ranged from 0.84 to 0.87 when deleting individual items, with all coefficients remaining ≥0.84. However, items Q6, Q10, and Q14 showed low item-total correlations of 0.14, 0.32, and 0.30, respectively (Appendix **4**).

### 3.6 Scale Construction

Based on the total and dimensional scores, percentiles and normative values were established for the healthcare service user sample, considering three levels: low (18-63), medium (64-79), and high (80-90).

Appendix **5** and Appendix **6** present the final questionnaire and normative values, respectively.

## 4 DISCUSSION

The aim of this study was to develop and validate an instrument for measuring eHealth literacy among health service users. The results suggest that the instrument has a four-dimensional factorial structure.

In exploratory factor analysis (EFA), commonly used methods for determining the number of factors include retaining those with an eigenvalue greater than one or using the scree plot [52]. The Kaiser rule (Eigenvalue > 1) is considered unreliable and outdated [60, 61].

Items with factor loadings below 0.30 are considered inadequate, as they contribute less than 10% of the variance in the measured latent construct [62]. The rules for retaining an item within a factor are generally arbitrary, and there is no mathematical test indicating that values must exceed |0.3| or |0.6|; instead, a strong rationale should be provided for the chosen threshold [60]. More complex, often simulation-based approaches exist that offer a more accurate estimation of the number of latent factors, and in this context, parallel analysis is considered the gold standard for factor retention [53, 61].

In this study, the Kaiser rule suggested three factors, while according to the graph curve it suggests four factors. We decided to retain four factors, which were then extracted using the Weighted Least Squares (WLS) method with Oblimin rotation.

The resulting factors were defined as follows: WLS1 (Q7, Q8, Q9, Q10, Q11, Q12, Q13), WLS4 (Q1, Q2, Q3, Q4, Q5), WLS3 (Q14, Q15, Q16), and WLS2 (Q6, Q17, Q18), collectively explaining 59.3% of the total variance. These factors were subsequently labeled as “Digital Literacy” (WLS1), “Digital Self-Management” (WLS4), “Digital Skill” (WLS3), and “Scientific Empowerment” (WLS2), respectively.

There is no established rule to guide the selection of an orthogonal or oblique rotation technique [51]. Among the various rotation methods available, Varimax is the most widely used orthogonal method [39]. In this study, we employed Oblimin rotation. This technique minimizes the cross-products of loadings to simplify factors, and it is also the preferred method when working with large datasets [63].

Factor rotation is intended to achieve a more interpretable solution [39]. Moreover, factor rotation does not alter the amount of total variance explained but facilitates the analytical process by more clearly associating each variable with a single factor and enhancing its explanatory power [51].

In this study, the four extracted factors included an adequate number of indicators. However, one item (Q6) showed a positive factor loading of 0.31 on Factor WLS2, while simultaneously presenting a negative loading of -0.31 on Factor WLS3.

When alternative extraction methods were applied [64]—such as Minimum Residuals (MINRES), Weighted Least Squares Mean and Variance Adjusted (WLSMV), and Unweighted Least Squares (ULS), all compatible with polychoric matrices—all items remained within the four-factor structure. Nonetheless, item Q6 had a higher positive loading (0.311) on the fourth factor, along with items Q17 and Q18 (while on the third factor, item Q6 showed a negative loading of -0.308). Therefore, we decided to retain item Q6 within Factor WLS2.

Several authors have noted that at least three measured variables are required for the statistical identification of a factor. Although more indicators are preferable, it is recommended to have four to six indicators per factor [39, 65]. A factor with fewer than three items is typically considered weak and unstable; however, with a very large dataset, it may be possible to reduce the number of items while still maintaining a strong factor [66]. The quality of the items is also considered as indicators of the factor [67].

There is a strong factor loading for each item associated with the four extracted factors. A factor loading greater than 0.30 generally indicates a moderate correlation between the item and the factor. Additionally, the higher the communality value (h^2^), the more the extracted factors explain the item's variance [68]. The remaining variance of the item is considered unique variance (u^2^), which does not contribute to the measurement of the common factors [65].

In the study, with the exception of three items belonging to three factors [WLS4 (Q2), WLS2 (Q6), and WLS1 (Q10)], the h^2^ values were greater than 0.5 (0.50 < h^2^ ≤ 0.99).

Regarding reliability, when working with the 21 items, the Cronbach's Alpha coefficient obtained was 0.85. However, for reliability within each factor, only one factor had a value of 0.83, while the remaining three factors had values lower than 0.70. The three eliminated items (q1, q7, and q11) had a significant impact on reliability.

When eliminating items, it is essential to conduct another EFA and report which variables were removed, as well as the criteria used to make the decision [64]. It is known that many items may be lost during the theoretical and psychometric analysis [48]. Therefore, the EFA results presented correspond to the data from the 18 items.

The new Cronbach's Alpha value for the complete 18-item scale was 0.86 (95% CI: 0.85-0.87), and 0.9 for McDonald's ω coefficient. Furthermore, the coefficients for each factor also improved [WLS1 (0.81, 95% CI: 0.80-0.83), WLS4 (0.72, 95% CI: 0.70-0.75), WLS3 (0.67, 95% CI: 0.64-0.71), and WLS2 (0.60, 95% CI: 0.56-0.64)]. After eliminating an item, the coefficients remained ≥ 0.84; however, items Q6, Q10, and Q14 had low item-total correlations of 0.14, 0.32, and 0.30, respectively. Items with very low adjusted item-total correlations (< 0.30) are less desirable and may indicate the need for their possible removal from the scale [62].

To meet the minimum required number of items per factor [39, 65], we decided to retain these items with low item-total correlations, but minor modifications in wording were made without substantially altering the content. For minor modifications, a formal assessment of the psychometric properties of the instrument may not be necessary, or a small-scale pilot test may suffice [69]. In our case, the change was made with the involvement of one of the experts. The way respondents interpret the meaning of the questions strongly influences their answers; therefore, it is important that the questions are brief, clear, and unambiguous [62, 70].

Regarding the Cronbach's alpha coefficient, there is a wide range of qualitative descriptors based on the value range [71], with acceptable alpha values considered between 0.70 and 0.95 [56], although values between 0.80 and 0.95 are preferred for the psychometric quality of scales [62]. A low alpha coefficient may be due to a low number of questions, poor inter-item correlations, or heterogeneous constructs, while a high value (>0.90) may suggest redundancies [56].

Although the alpha coefficient is often used as a measure of internal consistency and reliability [56-58], it has its limitations, as it can either underestimate or overestimate; in this regard, the omega coefficient (ω) provides a better estimate of an instrument's reliability than the alpha coefficient [57].

The alpha coefficient is lower than omega, and therefore, it can be trusted as the lower bound of reliability; moreover, the difference between alpha and omega has no practical consequences when the factor loadings average 0.70 [72]. For now, there is insufficient reason to completely abandon the use of alpha and replace it with omega, but rather to use both coefficients as complementary estimators [73].

We consider that the 18-item instrument has a reasonable length and meets the criteria for validity and reliability. There is no general rule for determining the number of items in a questionnaire; however, the questionnaire should contain enough items to measure the construct of interest, but it should not be so long that respondents experience fatigue or loss of motivation [46].

E-Health literacy considers various aspects. In this regard, the items grouped into the four factors obtained, namely “Digital Literacy,” “Digital Self-management,” “Digital Skill,” and “Scientific Empowerment,” are relevant and consistent with the “lily model” of eHealth literacy, which includes six types of literacy [8].

Digital literacy refers to the ability to find and use health information through technology [74], while digital self-management can help individuals manage their health issues [75]. On the other hand, digital skill involves the ability to use computers and adapt to new technologies [8], whereas scientific empowerment refers to patient participation in co-creating knowledge with the aim of achieving autonomy [76].

Within the limitations and strengths, one limitation is that the study population was limited to primary healthcare users from a single district; however, some of these users also visit at least three hospitals in the Callao Region. Additionally, neighboring districts have similar sociodemographic characteristics and also host a significant number of migrants from other parts of the country, so the results can be at least extrapolated to the Peruvian population. Furthermore, the neighboring districts have similar sociodemographic characteristics and also host a significant number of migrants from other regions of the country, so the results can be extrapolated at least to the Peruvian population.

It should also be considered as a limitation that the study did not include confirmatory factor analysis (CFA), which would have allowed for a more complete analysis of the psychometric properties of the instrument. CFA is planned for future studies, along with the possibility of validation in other contexts.

On the other hand, we consider the sample size as a strength, as it was much larger than the minimum recommended sample, which was crucial for the EFA based on a polychoric matrix. An EFA based on a polychoric correlation matrix requires a larger sample compared to a Pearson correlation matrix to achieve the same level of precision and stability [65].

## CONCLUSION

The results of this study support that the eHealth literacy assessment scale for healthcare service users shows adequate levels of validity and reliability. The findings suggest that the questionnaire could be considered as an alternative for use both at the primary care level and in the hospital setting. However, additional studies are recommended to expand the psychometric analysis.

It is also relevant to explore its performance in diverse populations and different cultural contexts, as well as its usefulness in interventions aimed at improving eHealth literacy. These actions will contribute to consolidating the instrument's robustness and optimizing its applicability in both clinical and community settings.

## Figures and Tables

**Appendix 1:** Average evaluation of items and Aiken coefficient.

N°	N°	Pertinence			Relevance			Clarity		
Item	Judges	Mean (SD)	V [95% CI]		Mean (SD)	V [95% CI]		Mean (SD)	V [95% CI]	
q1	12	4.00(0.00)	1.00 [0.90-1.00]		4.00(0.00)	1.00 [0.90-1.00]		4.00(0.00)	1.00 [0.90-1.00]	
q2	12	4.00(0.00)	1.00 [0.90-1.00]		4.00(0.00)	1.00 [0.90-1.00]		4.00(0.00)	1.00 [0.90-1.00]	
q3	12	3.92(0.29)	0.97 [0.86-1.00]		3.92(0.29)	0.97 [0.86-1.00]		3.92(0.29)	0.97 [0.86-1.00]	
q4	12	2.58(1.38)	0.53 [0.37-0.68]		2.58(1.38)	0.53 [0.37-0.68]		2.58(1.38)	0.53 [0.37-0.68]	
q5	12	3.92(0.29)	0.97 [0.86-1.00]		3.92(0.29)	0.97 [0.86-1.00]		3.92(0.29)	0.97 [0.86-1.00]	
q6	12	3.92(0.29)	0.97 [0.86-1.00]		3.92(0.29)	0.97 [0.86-1.00]		3.92(0.29)	0.97 [0.86-1.00]	
q7	12	3.92(0.29)	0.97 [0.86-1.00]		3.92(0.29)	0.97 [0.86-1.00]		3.92(0.29)	0.97 [0.86-1.00]	
q8	12	3.58(0.90)	0.86 [0.71-0.94]		3.58(0.90)	0.86 [0.71-0.94]		3.58(0.90)	0.86 [0.71-0.94]	
q9	12	3.92(0.29)	0.97 [0.86-1.00]		3.92(0.29)	0.97 [0.86-1.00]		3.92(0.29)	0.97 [0.86-1.00]	
q10	12	3.50(1.17)	0.83 [0.68-0.92]		3.50(1.17)	0.83 [0.86-0.92]		3.50(1.17)	0.83 [0.68-0.92]	
q11	12	3.83(0.58)	0.94 [0.82-0.98]		3.83(0.58)	0.94 [0.82-0.98]		3.83(0.58)	0.94 [0.82-0.98]	
q12	12	3.58(0.90)	0.86 [0.71-0.94]		3.58(0.90)	0.86 [0.71-0.94]		3.58(0.90)	0.86 [0.71-0.94]	
q13	12	3.92(0.29)	0.97 [0.86-1.00]		3.92(0.29)	0.97 [0.86-1.00]		3.92(0.29)	0.97 [0.86-1.00]	
q14	12	3.83(0.58)	0.94 [0.82-0.98]		3.83(0.58)	0.94 [0.82-0.98]		3.83(0.58)	0.94 [0.82-0.98]	
q15	12	4.00(0.00)	1.00 [0.90-1.00]		4.00(0.00)	1.00 [0.90-1.00]		4.00(0.00)	1.00 [0.90-1.00]	
q16	12	3.83(0.58)	0.94 [0.82-0.98]		3.83(0.58)	0.94 [0.82-0.98]		3.83(0.58)	0.94 [0.82-0.98]	
q17	12	3.92(0.29)	0.97 [0.86-1.00]		3.92(0.29)	0.97 [0.86-1.00]		3.92(0.29)	0.97 [0.86-1.00]	
q18	12	3.83(0.39)	0.94 [0.82-0.98]		3.83(0.39)	0.94 [0.82-0.98]		3.83(0.39)	0.94 [0.82-0.98]	
q19	12	3.92(0.29)	0.97 [0.86-1.00]		3.92(0.29)	0.97 [0.86-1.00]		3.92(0.29)	0.97 [0.86-1.00]	
q20	12	3.83(0.39)	0.94 [0.82-0.98]		3.83(0.39)	0.94 [0.82-0.98]		3.83(0.39)	0.94 [0.82-0.98]	
q21	12	4.00(0.00)	1.00 [0.90-1.00]		4.00(0.00)	1.00 [0.90-1.00]		4.00(0.00)	1.00 [0.90-1.00]	
q22	12	3.92(0.29)	0.97 [0.86-1.00]		3.92(0.29)	0.97 [0.86-1.00]		3.92(0.29)	0.97 [0.86-1.00]	
q23	12	3.67(0.89)	0.89 [0.75-0.96]		3.67(0.89)	0.89 [0.75-0.96]		3.67(0.89)	0.89 [0.75-0.96]	
q24	12	4.00(0.00)	1.00 [0.90-1.00]		4.00(0.00)	1.00 [0.90-1.00]		4.00(0.00)	1.00 [0.90-1.00]	
q25	12	4.00(0.00)	1.00 [0.90-1.00]		4.00(0.00)	1.00 [0.90-1.00]		4.00(0.00)	1.00 [0.90-1.00]	
q26	12	4.00(0.00)	1.00 [0.90-1.00]		4.00(0.00)	1.00 [0.90-1.00]		4.00(0.00)	1.00 [0.90-1.00]	
q27	12	3.92(0.29)	0.97 [0.86-1.00]		3.92(0.29)	0.97 [0.86-1.00]		3.92(0.29)	0.97 [0.86-1.00]	
q28	12	3.83(0.58)	0.94 [0.82-0.98]		3.83(0.58)	0.94 [0.82-0.98]		3.83(0.58)	0.94 [0.82-0.98]	
q29	12	3.92(0.29)	0.97 [0.86-1.00]		3.92(0.29)	0.97 [0.86-1.00]		3.92(0.29)	0.97 [0.86-1.00]	
q30	12	4.00(0.00)	1.00 [0.90-1.00]		4.00(0.00)	1.00 [0.90-1.00]		4.00(0.00)	1.00 [0.90-1.00]	
q31	12	3.67(0.89)	0.89 [0.75-0.96]		3.67(0.89)	0.89 [0.75-0.96]		3.67(0.89)	0.89 [0.75-0.96]	
q32	12	3.83(0.58)	0.94 [0.82-0.98]		3.83(0.58)	0.94 [0.82-0.98]		3.83(0.58)	0.94 [0.82-0.98]	
q33	12	3.83(0.39)	0.94 [0.82-0.98]		3.83(0.39)	0.94 [0.82-0.98]		3.83(0.39)	0.94 [0.82-0.98]	
Domain		0.94			0.94			0.92		
Global		0.93 [95% CI: 0.79-0.97]								

**Appendix 2:** Descriptive statistics of the instrument.

Item	n	mean	sd	median	trimmed	mad	min	max	range	skew	kurtosis	se
q1	1068	4.96	0.28	5	5.00	0.00	1	5	4	-9.53	108.90	0.01
q2	1068	4.59	0.75	5	4.74	0.00	1	5	4	-2.63	8.40	0.02
q3	1068	3.64	1.50	4	3.80	1.48	1	5	4	-0.77	-0.92	0.05
q4	1068	4.24	1.00	4	4.45	1.48	1	5	4	-1.62	2.35	0.03
q5	1068	4.11	1.21	4	4.37	1.48	1	5	4	-1.51	1.28	0.04
q6	1068	4.56	0.81	5	4.75	0.00	1	5	4	-2.26	5.35	0.02
q7	1068	3.60	1.54	4	3.75	1.48	1	5	4	-0.81	-0.93	0.05
q8	1068	1.80	1.34	1	1.50	0.00	1	5	4	1.54	0.87	0.04
q9	1068	3.86	1.44	4	4.07	1.48	1	5	4	-0.98	-0.53	0.04
q10	1068	3.60	1.58	4	3.75	1.48	1	5	4	-0.71	-1.15	0.05
q11	1068	2.96	1.66	3	2.95	2.97	1	5	4	0.01	-1.70	0.05
q12	1068	3.06	1.75	4	3.07	1.48	1	5	4	-0.10	-1.77	0.05
q13	1068	4.71	0.50	5	4.78	0.00	1	5	4	-1.93	6.46	0.02
q14	1068	3.89	1.29	4	4.09	1.48	1	5	4	-1.06	-0.12	0.04
q15	1068	4.45	0.92	5	4.66	0.00	1	5	4	-1.97	3.70	0.03
q16	1068	4.32	0.99	5	4.54	0.00	1	5	4	-1.61	1.96	0.03
q17	1068	4.30	1.19	5	4.59	0.00	1	5	4	-1.81	2.07	0.04
q18	1068	4.63	0.75	5	4.79	0.00	1	5	4	-2.64	8.04	0.02
q19	1068	4.63	0.81	5	4.82	0.00	1	5	4	-2.78	8.00	0.02
q20	1068	3.02	1.40	3	3.03	1.48	1	5	4	-0.21	-1.33	0.04
q21	1068	2.77	1.40	3	2.71	1.48	1	5	4	0.19	-1.32	0.04

**Appendix 3:** Polychoric correlation matrix of the items.

	Q1	Q2	Q3	Q4	Q5	Q6	Q7	Q8	Q9	Q10	Q11	Q12	Q13	Q14	Q15	Q16	Q17	Q18
Q1	1	0.526	0.575	0.530	0.573	0.015	0.561	0.626	0.465	0.360	0.492	0.506	0.498	0.397	0.656	0.470	0.293	0.238
Q2	0.526	1	0.501	0.471	0.434	0.058	0.498	0.528	0.409	0.248	0.505	0.461	0.386	0.208	0.454	0.371	0.309	0.267
Q3	0.575	0.501	1	0.601	0.631	0.151	0.501	0.583	0.397	0.325	0.553	0.562	0.500	0.208	0.556	0.313	0.286	0.223
Q4	0.530	0.471	0.601	1	0.564	0.057	0.531	0.551	0.446	0.262	0.592	0.472	0.430	0.319	0.563	0.391	0.280	0.249
Q5	0.573	0.434	0.631	0.564	1	0.175	0.604	0.643	0.504	0.290	0.568	0.617	0.471	0.336	0.649	0.443	0.323	0.262
Q6	0.015	0.058	0.151	0.057	0.175	1	0.048	0.050	0.029	-0.024	0.095	0.069	0.043	-0.172	0.026	-0.042	0.214	0.208
Q7	0.561	0.498	0.501	0.531	0.604	0.048	1	0.857	0.623	0.335	0.628	0.602	0.605	0.304	0.627	0.462	0.348	0.260
Q8	0.626	0.528	0.583	0.551	0.643	0.050	0.857	1	0.697	0.324	0.668	0.652	0.666	0.327	0.649	0.456	0.351	0.275
Q9	0.465	0.409	0.397	0.446	0.504	0.029	0.623	0.697	1	0.290	0.583	0.533	0.576	0.314	0.604	0.513	0.357	0.281
Q10	0.360	0.248	0.325	0.262	0.290	-0.024	0.335	0.324	0.290	1	0.326	0.270	0.366	0.176	0.412	0.312	0.210	0.192
Q11	0.492	0.505	0.553	0.592	0.568	0.095	0.628	0.668	0.583	0.326	1	0.570	0.598	0.244	0.575	0.370	0.272	0.222
Q12	0.506	0.461	0.562	0.472	0.617	0.069	0.602	0.652	0.533	0.270	0.570	1	0.530	0.273	0.569	0.359	0.309	0.234
Q13	0.498	0.386	0.500	0.430	0.471	0.043	0.605	0.666	0.576	0.366	0.598	0.530	1	0.193	0.541	0.296	0.292	0.213
Q14	0.397	0.208	0.208	0.319	0.336	-0.172	0.304	0.327	0.314	0.176	0.244	0.273	0.193	1	0.543	0.619	0.222	0.159
Q15	0.656	0.454	0.556	0.563	0.649	0.026	0.627	0.649	0.604	0.412	0.575	0.569	0.541	0.543	1	0.683	0.420	0.333
Q16	0.470	0.371	0.313	0.391	0.443	-0.042	0.462	0.456	0.513	0.312	0.370	0.359	0.296	0.619	0.683	1	0.418	0.319
Q17	0.293	0.309	0.286	0.280	0.323	0.214	0.348	0.351	0.357	0.210	0.272	0.309	0.292	0.222	0.420	0.418	1	0.749
Q18	0.238	0.267	0.223	0.249	0.262	0.208	0.260	0.275	0.281	0.192	0.222	0.234	0.213	0.159	0.333	0.319	0.749	1

**Appendix 4:** Reliability if an item is dropped.

Item	n	raw_alpha	std.alpha	raw.r	r.cor
Q1	1068	0.85	0.86	0.57	0.58
Q2	1068	0.85	0.87	0.56	0.49
Q3	1068	0.85	0.86	0.61	0.60
Q4	1068	0.85	0.87	0.58	0.54
Q5	1068	0.85	0.86	0.63	0.65
Q6	1068	0.87	0.88	0.24	0.14
Q7	1068	0.84	0.86	0.73	0.71
Q8	1068	0.84	0.86	0.77	0.75
Q9	1068	0.85	0.86	0.66	0.59
Q10	1068	0.86	0.87	0.33	0.32
Q11	1068	0.85	0.86	0.65	0.62
Q12	1068	0.85	0.86	0.61	0.59
Q13	1068	0.85	0.87	0.55	0.52
Q14	1068	0.86	0.87	0.34	0.30
Q15	1068	0.85	0.86	0.67	0.70
Q16	1068	0.85	0.87	0.53	0.53
Q17	1068	0.86	0.87	0.53	0.48
Q18	1068	0.86	0.87	0.50	0.44

**Appendix 5:** Final eHealth literacy questionnaire.

ITEM	ANNOUNCEMENT	1	2	3	4	5
	**Digital literacy**					
1	I can search for and find information online about the services offered by a healthcare facility [Puedo buscar y encontrar en internet los servicios que ofrece un establecimiento de salud (Q7)].					
2	I can request information online or through apps about the services of a healthcare facility [Puedo solicitar información a través de internet o sus aplicativos sobre los servicios de un establecimiento de salud (Q8)].					
3	I know how to receive medical prescriptions or instructions from a healthcare professional via email or a digital application [Sé cómo recibir recetas médicas o indicaciones del profesional de la salud a través de correo electrónico o alguna aplicación digital (Q9)].					
4	I can read and understand the instructions received from the healthcare professional [Puedo leer y entender las indicaciones recibidas del profesional de la salud (Q10)].					
5	I can consult and resolve my doubts with a professional based on the information I find on the internet or social media [Puedo consultar y resolver mis dudas con un profesional, basándome en la información que encuentro en Internet o en las redes sociales (Q11)].					
6	I take into account the instructions and recommendations provided by healthcare institutions through the internet or social media [Tomo en cuenta las indicaciones y recomendaciones ofrecidas por las instituciones sanitarias a través de internet o redes sociales (Q12)].					
7	I am well informed about my health problem and feel empowered to make decisions together with the healthcare professional [Estoy bien informado sobre mi problema de salud y me siento capacitado para tomar decisiones en conjunto con el profesional de la salud (Q13)].					
	**Digital self-management**					
8	I can search for information on the internet about any health-related topic [Puedo buscar información en internet sobre cualquier tema relacionado con la salud (Q1)].					
9	I can find information on health topics published on the websites of healthcare institutions [Puedo encontrar información sobre temas de salud publicada en la página web de las instituciones sanitarias (Q2)].					
10	I use the information found on the internet to improve or maintain my health [Utilizo la información encontrada en internet para mejorar o conservar mi estado de salud (Q3)].					
11	I search for information on the internet to complement what the healthcare professional provides me during the consultation [Busco información en internet para complementar lo que el profesional de la salud me proporciona durante la consulta (Q4)].					
12	I can learn about health topics by searching on social media or the internet [Puedo aprender sobre temas de salud buscando en redes sociales o internet (Q5)].					
	**Digital skills**					
13	I have the skills to use a computer or any digital device [Tengo habilidades para usar computadora o cualquier dispositivo digital (Q14)].					
14	I have the skills to search for information on the internet or its applications about any health topic that interests me [Tengo habilidades para buscar información en internet o en sus aplicaciones sobre cualquier tema de salud que me interesa (Q15)].					
15	I can communicate via email, text messaging, social media, and video calls [Puedo comunicarme por correo electrónico, mensajería de texto, redes sociales y videollamadas (Q16)].					
	**Scientific empowerment**					
16	I take care of my health based on information available on the social media of a healthcare professional [Cuido mi salud basado en información disponible en las redes sociales de algún profesional de la salud (Q6)].					
17	I can easily read and understand scientific information [Puedo leer y comprender con facilidad información científica (Q17)].					
18	I can tell whether health information is scientific or not [Puedo diferenciar si una información sobre salud es científica o no (Q18)].					

(*) 1: Strongly Disagree, 2: Somewhat Disagree, 3: Neither Agree nor Disagree, 4: Somewhat agree, and 5: Strongly agree.

**Appendix 6:** Construction of scales.

	Global score	Score by dimension			
	(Q1 to Q18)	WLS1	WLS4	WLS3	WLS1
Min.	18	7	5	3	3
Max.	90	35	25	15	15
Percentile					
5	48	17	14	9	3
10	54	19	16	10.7	3
15	58	20	18	11	4
20	61	22	19	12	5
25	63	23	19	13	5
30	66	25	20	14	6
35	67	26	20	14	6
40	69	27	21	14	7
45	71	28	21	14	7
50	72	29	22	15	7
55	73	30	22	15	8
60	75	31	23	15	8
65	76	31	23.5	15	9
70	77	33	24	15	9
75	79	34	24	15	9
80	88	34	25	15	10
85	82	35	25	15	11
90	84	35	25	15	11
95	86	35	25	15	14
100	90	35	25	15	15
Classification					
Low	18-63	7-23	5-19	3-13	3-5
Medium	64-79	24- 33	20-23	14	6-9
High	80-90	34-35	24-25	15	10-15

**Table 1:** Sample Adequacy Measures for Exploratory Factor Analysis (EFA).

Sample Adequacy Measures for Exploratory Factor Analysis									Interpretation
Kaiser-Meyer-Olkin factor adequacy									
Overall MSA= 0.92									Adequate
MSA for each item:									
Q1	Q2	Q3	Q4	Q5	Q6	Q7	Q8	Q9	
0.96	0.95	0.93	0.95	0.96	0.66	0.91	0.90	0.95	
Q10	Q11	Q12	Q13	Q14	Q15	Q16	Q17	Q18	
0.94	0.96	0.97	0.95	0.86	0.95	0.89	0.80	0.76	
Bartlett test of homogeneity of variances									
Bartlett's K-squared = 11750.31, df = 153, p-value < 0.001									Adequate for EFA

**Table 2:** Factor Analysis with four Components.

-	WLS1	WLS4	WLS3	WLS2	h2	u2	com
Q1	0.167	**0.457**	0.288	-0.017	0.575	0.425	1.99
Q2	0.218	**0.379**	0.056	0.093	0.395	0.605	1.79
Q3	-0.025	**0.866**	-0.026	0.028	0.718	0.282	1.01
Q4	0.104	**0.563**	0.148	0.018	0.527	0.473	1.21
Q5	0.183	**0.552**	0.128	0.052	0.617	0.383	1.36
Q6	-0.086	0.249	-0.310	**0.310**	0.159	0.841	3.06
Q7	**0.882**	-0.026	0.016	0.011	0.768	0.232	1.00
Q8	**0.947**	0.009	-0.018	-0.008	0.885	0.115	1.00
Q9	**0.723**	-0.099	0.136	0.075	0.590	0.410	1.13
Q10	**0.181**	0.154	0.150	0.048	0.186	0.814	3.08
Q11	**0.533**	0.315	-0.026	-0.008	0.601	0.399	1.63
Q12	**0.445**	0.327	0.016	0.024	0.542	0.458	1.85
Q13	**0.686**	0.108	-0.076	0.011	0.539	0.461	1.07
Q14	-0.077	0.023	**0.797**	-0.057	0.563	0.437	1.03
Q15	0.205	0.300	**0.511**	0.076	0.777	0.223	2.04
Q16	0.055	-0.011	**0.766**	0.142	0.729	0.271	1.08
Q17	0.032	-0.024	0.032	**0.900**	0.840	0.160	1.01
Q18	-0.038	0.018	-0.015	**0.831**	0.670	0.330	1.01

(*) Extraction method: Weighted Least Squares (WLS), Rotation method: Oblimin, h2: Communality, u2: Uniqueness, com: Complexity.

**Table 3:** Total Variance Explained.

	WLS1	WLS4	WLS3	WLS2
SS loadings	4.030	2.819	2.065	1.769
Proportion Var	0.224	0.157	0.115	0.098
Cumulative Var	0.224	0.381	0.495	0.593
Proportion Explained	0.377	0.264	0.193	0.166
Cumulative Proportion	0.377	0.641	0.834	1.000

**Table 4:** Reliability Analysis.

Number of items (n=18)	Reliability Statistics	
	Cronbach´s Alpha	McDonald´s ω
Global reliability	0.86 (0.85-0.87)	0.9
Reliability by factors		
WLS1 (Q7, Q8,Q9,Q10,Q11,Q12,Q13)	0.81 (0.80-0.83)	0.85
WLS4 (Q1,Q2,Q3,Q4,Q5)	0.72(0.70-0.75)	0.78
WLS3 (Q14,Q15,A16)	0.67(0.64-0.71)	0.72
WLS2 (Q6,Q17,Q18)	0.60(0.56-0.64)	0.68

## Data Availability

The data that support the findings of this study are available from the corresponding author [J.M.] on special request. It is also available at the following address: https://doi.org/10.5281/zenodo.15328068.

## LIST OF ABBREVIATIONS

EFA: = Exploratory factor analysis.
CFA: = Confirmatory Factor Analysis.
KMO: = Kaiser-Meyer-Olkin.
ICT: = Information and communication technologies.
EHEALS: = eHealth Literacy Scale.
WWW: = World Wide Web.
PDCA: = Plan-Do-Check-Act.
DEFF: = Design Effect.
Q1: = 25th percentile.
Q3: = 75th percentile.
IQR: = interquartile range.
AOR: = Adjusted Odds Ratio.
95%CI: = 95% confidence interval.
SD: = Standard Deviation.
SE: = Standard Error.
WLS: = Weighted Least Squares.
h2: = Communality
u2: = Uniqueness
com: = Complexity.

## STANDARDS OF REPORTING

STROBE guidelines were followed.
